# Precision Trajectory Tracking of Magnetically Actuated Microparticles Using Permanent-Magnet Systems Under State Constraints

**DOI:** 10.3390/mi17101177

**Published:** 2026-10-11

**Authors:** Zakaria El Hamdaoui, Siyuan Wang, Driss Boutat, Noureddine El Barbri, Karim Rhofir

**Affiliations:** 1 Institut National des Sciences Appliquées (INSA) Centre Val de Loire, University of Orléans, PRISME EA 4229, 18022 Bourges, France; siyuan.wang@insa-cvl.fr (S.W.); driss.boutat@insa-cvl.fr (D.B.); 2 Laboratoire LaSTI, École Nationale des Sciences Appliquées (ENSA) Khouribga, University Sultan Moulay Slimane, Khouribga 25000, Morocco; n.elbarbri@usms.ma (N.E.B.); k.rhofir@usms.ma (K.R.)

**Keywords:** magnetic microparticles, permanent-magnet actuation, precision trajectory tracking, state constraints, disturbance rejection, homogeneous control

## Abstract

Magnetically actuated microparticles are promising candidates for minimally invasive microrobotics and targeted delivery in confined microenvironments. Their precise navigation remains challenging because viscous drag, flow disturbances, and safety constraints can prevent accurate trajectory tracking. This paper investigates precision tracking of magnetic microparticles actuated by permanent-magnet systems under state constraints and bounded disturbances. The control problem is formulated at the magnetic-force level, where the force applied to the microparticle is treated as the controlled input. This formulation is motivated by permanent-magnet actuation architectures while remaining applicable when the desired magnetic force can be generated or approximated. An invariant-set-based disturbance rejection strategy is developed. First, an optimized linear controller is obtained using linear matrix inequalities (LMIs) to minimize the output invariant ellipsoid while satisfying state constraints. Then, a homogeneous controller is introduced to improve convergence and tracking accuracy. A worst-case disturbance direction is also characterized. Simulations in a blood-like viscous environment show improved convergence and accuracy with the homogeneous controller, supporting force-level constrained control for precision permanent-magnet actuation of microparticles.

## 1. Introduction

Magnetic microrobotics has attracted considerable attention for minimally invasive biomedical applications because magnetic fields enable wireless and contactless manipulation at the microscale. Magnetic microparticles can be remotely guided without onboard power sources and have been investigated for applications including targeted drug delivery, localized therapy, cell manipulation, and minimally invasive interventions [1,2,3,4].

Magnetic manipulation systems are generally based on electromagnetic coils or permanent magnets [5,6]. Electromagnetic systems offer programmable field generation but may require relatively bulky hardware, continuous electrical power, and thermal management. Permanent magnets, in contrast, can generate strong magnetic fields without continuous power consumption and provide compact and energy-efficient actuation solutions [7,8]. However, their nonlinear magnetic-field distribution and dependence on magnet position and orientation make precise microparticle control challenging.

Magnetic actuation of biomedical microrobots can rely on different physical mechanisms depending on their morphology and intended application. Rotating magnetic fields generate magnetic torques and are widely used to propel helical and other torque-driven microrobots, whereas spatial magnetic-field gradients can exert translational forces on magnetically responsive particles. These approaches address different actuation requirements and may offer different advantages depending on the operating conditions. In particular, permanent-magnet-based gradient actuation has been investigated for the manipulation of magnetic microbeads in viscous environments, with both numerical and experimental demonstrations reported in previous studies [9,10,11]. The present work focuses on this force-driven approach and investigates robust trajectory tracking of spherical magnetic microparticles under state constraints and bounded disturbances.

In biomedical and microfluidic environments, magnetic microparticles are also affected by viscous drag, flow disturbances, modeling uncertainties, and physical constraints. Consequently, accurate trajectory tracking must be achieved while maintaining the microparticle within an admissible region around the prescribed trajectory. State-constrained and robust control methods are therefore particularly relevant for magnetic microparticle navigation. Invariant-set and LMI-based techniques provide systematic tools for handling bounded disturbances and state constraints [12,13,14].

Previous work investigated the control of a two-magnet system under state and input constraints [15], as well as trajectory tracking of a magnetic microparticle using a dual-magnet system under state constraints [16]. Building on these results, the present work develops a constrained force-level control framework for magnetic microparticles actuated by permanent-magnet systems. The controller is formulated directly in terms of the magnetic force applied to the microparticle rather than for a particular permanent-magnet geometry, allowing different actuation configurations to be considered within the same control formulation.

Two control strategies are developed. First, an optimized linear controller based on invariant ellipsoids and LMIs is designed to reduce the effect of bounded disturbances while satisfying prescribed state constraints. The linear design is then extended using homogeneous control to improve convergence and tracking performance. The main contributions are summarized as follows:

- A force-level constrained control formulation applicable to permanent-magnet actuation systems;
- An LMI-based linear controller combining disturbance rejection with prescribed state constraints;
- A homogeneous extension preserving the invariant-set formulation while improving convergence performance;
- A comparison of the linear and homogeneous controllers for magnetic microparticle trajectory tracking in a viscous environment.

The remainder of this paper is organized as follows. Section 2 presents the permanent-magnet actuation principle and the magnetic microparticle model. Section 3 recalls the required homogeneous-control and invariant-set concepts. Section 4 develops the constrained linear and homogeneous controllers. Section 5 presents the simulation results, and Section 6 concludes the paper.

The following notation will be used throughout this paper.

R denotes the set of real numbers; ∥·∥ denotes the Euclidean norm in $R^{n}$, with $∥x∥=\sqrt{x^{⊤}x}$; and ${∥\cdot ∥}_{P}$ denotes the weighted norm in $R^{n}$, with ${∥x∥}_{P}=\sqrt{x^{⊤}Px}$. For a symmetric matrix $P\in R^{n\times n}$, P⪯0 (P⪰0) denotes that *P* is negative (positive) semidefinite, whereas P≺0 (P≻0) denotes that *P* is negative (positive) definite. Moreover, $\lambda_{\min}\left(P\right)$ and $\lambda_{\max}\left(P\right)$ denote the minimum and maximum eigenvalues of *P*, respectively, and tr(P) denotes the trace of *P*. For P⪰0, the square root of *P* is denoted by $P^{1/2}$ and satisfies ${\left(P^{1/2}\right)}^{2}=P$. The Schur complement lemma is extensively used in the following sections.

## 2. Permanent-Magnet Actuation Systems and Microparticle Modeling

### 2.1. Permanent-Magnet Actuation Systems

Permanent-magnet systems provide a compact and energy-efficient solution for the remote manipulation of magnetic microparticles. In this work, a two-permanent-magnet configuration is used to illustrate the actuation principle. The control framework developed subsequently is nevertheless formulated at the magnetic-force level and is therefore not restricted to this particular actuator geometry.

**Figure 1:**
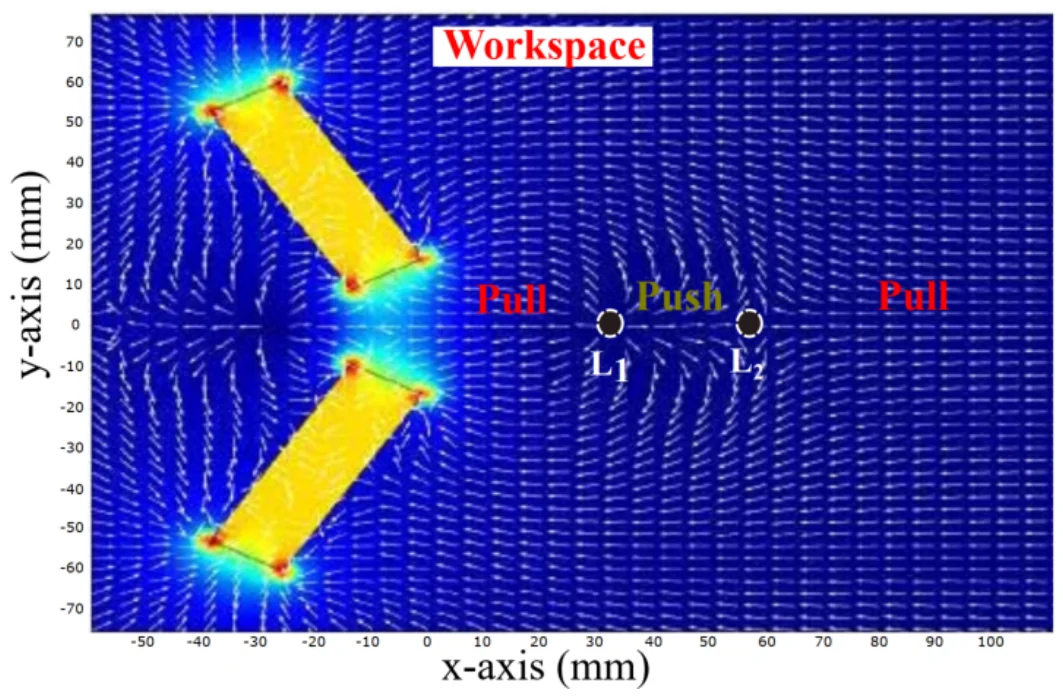
Permanent-magnet actuation system composed of two rectangular permanent magnets, represented by the yellow rectangles.

The two-magnet configuration consists of two rectangular permanent magnets and generates attractive and repulsive magnetic forces, as illustrated in Figure 1. Two characteristic equilibrium points can be identified: $L_{1}$ is an unstable equilibrium point, whereas $L_{2}$ is a stable equilibrium point toward which the magnetic-force vectors converge. This push–pull behavior can be exploited for magnetic microparticle manipulation [15,16].

More general permanent-magnet architectures, including configurations composed of multiple magnets, can also be considered to extend the achievable magnetic-field distribution and manipulation workspace. For an arrangement composed of $N_{m}$ permanent magnets, the total magnetic field can be expressed, according to the superposition principle, as

(1) $$B_{\mathrm{tot}}\left(x,y,z\right)=\sum_{j=1}^{N_{m}}B_{j}\left(x,y,z\right),$$

or, equivalently,

(2) $$B_{\mathrm{tot}}={\left[\sum_{j=1}^{N_{m}}B_{x,j}\;\sum_{j=1}^{N_{m}}B_{y,j}\;\sum_{j=1}^{N_{m}}B_{z,j}\right]}^{⊤},$$

where $B_{j}$ denotes the magnetic field generated by the *j*th permanent magnet.

For a uniformly magnetized rectangular permanent magnet, the three components of the magnetic field can be expressed analytically [17] as

$$\begin{array}{cc} B_{x}\left(x\right) & =\frac{M}{4\pi }\vartheta^{\left(ijk\right)}\left\{-\log\left(y-y_{j}+\delta_{ijk}\right)\right\},\\ B_{y}\left(x\right) & =\frac{M}{4\pi }\vartheta^{\left(ijk\right)}\left\{-\log\left(x-x_{i}+\delta_{ijk}\right)\right\},\\ B_{z}\left(x\right) & =\frac{M}{4\pi }\vartheta^{\left(ijk\right)}\left\{\arctan\left[\frac{\left(x-x_{i}\right)\left(y-y_{j}\right)}{\left(z-z_{k}\right)\delta_{ijk}}\right]\right\}, \end{array}$$

where $\delta_{ijk}=\sqrt{{\left(x-x_{i}\right)}^{2}+{\left(y-y_{j}\right)}^{2}+{\left(z-z_{k}\right)}^{2}}$, and $\vartheta^{\left(ijk\right)}\left\{f_{ijk}\right\}$ denotes the alternating sum $\sum_{i,j,k=1}^{2}{\left(-1\right)}^{i+j+k}f_{ijk}$ over the magnet corners. Here, *M* denotes the magnetization of the permanent magnet and $x_{i}$, $y_{j}$, and $z_{k}$ denote the coordinates of its faces in the local reference frame. The fields of multiple magnets can then be transformed into a common reference frame and superposed as in Equations (1) and (2); Equation (5) relates the resulting field to the force acting on the microparticle.

Regardless of the specific permanent-magnet arrangement, the control framework developed in this work is formulated at the magnetic-force level. The effect of the magnetic actuation system on the microparticle is represented by the resultant magnetic-force vector

(3) $$F_{m}={\left[F_{x}\;F_{y}\;F_{z}\right]}^{⊤}.$$

The controller developed in this work therefore determines the magnetic force required for trajectory tracking rather than directly controlling the individual positions or orientations of the permanent magnets. This force-level representation separates the control design from the specific actuator geometry and allows the proposed framework to be considered for different permanent-magnet configurations, provided that the commanded force lies within the achievable actuation capabilities of the system.

### 2.2. Physical Modeling of Magnetic Microparticles

Consider a spherical magnetic microparticle navigating in a viscous fluid environment under the action of an external permanent-magnet actuation system. The motion of the microparticle may be influenced by several physical effects, including magnetic forces, hydrodynamic drag, inertia, gravity, buoyancy, Brownian motion, electrical double-layer interactions, and Van der Waals forces. Among these contributions, the magnetic, hydrodynamic, gravitational, and buoyancy forces are considered dominant in the dynamic model, whereas the remaining effects are neglected [18].

The magnetic force generated by an external magnetic field acting on a magnetic object with magnetization m and volume $V_{m}$ is expressed as [19]

(4) $$F_{m}=\int_{V_{m}}\left(m\cdot ▿\right)BdV_{m},$$

where $B=B_{\mathrm{tot}}$ denotes the total magnetic field generated by the permanent-magnet actuation system. Assuming that the magnetization of the magnetic microparticle is uniform and constant, denoted by M, the magnetic force can be simplified as

(5) $$F_{m}=V_{m}\left(M\cdot ▿\right)B.$$

Equation (5) establishes the direct relationship between the magnetic-field gradient generated by the permanent-magnet actuation system and the resulting force applied to the magnetic microparticle. This relationship provides the physical connection between the actuation principle introduced in the previous subsection and the force-level control formulation developed subsequently.

The hydrodynamic behavior of a microparticle moving in a fluid depends strongly on the Reynolds number. In the present work, the motion is considered in the low-Reynolds-number regime, Re≪1, which is representative of microscale motion in highly viscous or confined fluidic environments. Under this assumption, the hydrodynamic drag acting on the spherical magnetic microparticle can be described using Stokes’ law as

(6) $$F_{t}=-6\pi \alpha r\left(v-v_{f}\right),$$

where α denotes the dynamic viscosity of the fluid, *r* is the radius of the magnetic microparticle, v is its velocity, and $v_{f}$ represents the local fluid velocity.

In addition to magnetic and hydrodynamic forces, a magnetic microparticle immersed in a fluid is subjected to gravity and buoyancy. The gravitational force is expressed as

(7) $$F_{g}=\rho Vg,$$

where *V* and ρ denote the volume and density of the magnetic microparticle, respectively, and g is the gravitational acceleration vector. The buoyancy force is given by

(8) $$F_{b}=-\rho_{f}Vg,$$

where $\rho_{f}$ denotes the fluid density. Therefore, the resultant gravitational and buoyancy force can be written as

(9) $$F_{gb}=\left(\rho -\rho_{f}\right)Vg.$$

By applying Newton’s second law, the translational dynamics of the magnetic microparticle are consequently described by

(10) $$m\ddot{x}=F_{m}+F_{t}+F_{gb}+\omega ,$$

where *m* denotes the mass of the magnetic microparticle, $x=(x_{n},y_{n},z_{n})$ is its position vector, and ω represents unknown external disturbances and modeling uncertainties.

The disturbance is assumed to be bounded according to

(11) $${\left||\omega |\right|}_{Q}\leq 1,\;Q=Q^{⊤}≻0.$$

This dynamic representation forms the basis of the state-space model and the constrained trajectory-tracking problem introduced in the following subsection.

### 2.3. State-Space Representation and Tracking Problem

Based on the dynamic model established in the previous subsection, the motion of the magnetic microparticle can be represented in state-space form. By considering the magnetic force $F_{m}$ as the actuation input and incorporating the hydrodynamic, gravitational, and buoyancy contributions, the dynamic model in (10) can be written as

(12) $$\begin{array}{cc} \; & \dot{\xi }=A\xi +B\left(F_{m}+G\right)+{D\omega ,\;||\omega ||}_{Q}\leq 1, \end{array}$$

(13) $$\begin{array}{cc} \; & y=C\xi , \end{array}$$

where the state vector is defined as

(14) $$\xi ={\left[x,\dot{x}\right]}^{⊤},$$

with $x=(x_{n},y_{n},z_{n})$ denoting the position of the magnetic microparticle. The output matrix is given by

(15) $$C=\left[I,0\right]\in R^{3\times 6},$$

where

(16) $$I=\left(\begin{array}{ccc} 1 & 0 & 0\\ 0 & 1 & 0\\ 0 & 0 & 1 \end{array}\right).$$

The system matrices are defined as

$$\begin{array}{cc} A & =\left(\begin{array}{cc} 0 & I\\ 0 & -\frac{6\pi \alpha r}{m}I \end{array}\right)\in R^{6\times 6},\\ B & =\left(\begin{array}{c} 0\\ \frac{1}{m}I \end{array}\right)\in R^{6\times 3}, \end{array}$$

and

$$\begin{array}{cc} G & =\left(6\pi \alpha rv_{f}+F_{gb}\right)\in R^{3\times 1},\\ D & =\left(\begin{array}{c} 0\\ I \end{array}\right)\in R^{6\times 3}. \end{array}$$

In this representation, *G* accounts for the contribution of the fluid velocity together with the resultant gravitational and buoyancy force, whereas ω represents the bounded external disturbance and modeling uncertainties. Importantly, $F_{m}$ constitutes the magnetic-force input generated by the permanent-magnet actuation system. Therefore, the state-space model provides a force-level representation that is independent of a specific permanent-magnet actuator geometry.

The control objective is to make the magnetic microparticle follow a prescribed reference trajectory. The reference dynamics are defined by

(17) $$\begin{array}{cc} {\dot{\xi }}_{r}\left(t\right) & =A\xi_{r}\left(t\right)+Bu_{0}\left(t\right), \end{array}$$

(18) $$\begin{array}{cc} y_{r}\left(t\right) & =C\xi_{r}\left(t\right), \end{array}$$

where $\xi_{r}\left(t\right)$ and $y_{r}\left(t\right)$ denote the reference state and reference output, respectively, and $u_{0}\left(t\right)$ is the corresponding reference input.

Defining the state tracking error, control variable, and output tracking error as

(19) $$\sigma =\xi -\xi_{r},$$

(20) $$u=F_{m}-u_{0}+G,$$

and

(21) $$y_{e}=y-y_{r},$$

respectively, the tracking-error dynamics can be expressed as

(22) $$\begin{array}{cc} \dot{\sigma }\left(t\right)\; & =A\sigma (t)+Bu(t)+D\omega (t),\\ y_{e}\left(t\right)\; & =C\sigma (t). \end{array}$$

In practical magnetic microparticle navigation, accurate trajectory tracking alone is not sufficient. The microparticle must also remain within an admissible region around the prescribed trajectory. Such a requirement is particularly important in confined environments, where excessive tracking errors may lead the microparticle outside the desired navigation region. Accordingly, the components of the tracking error are constrained as

(23) $$\left|\right|\sigma_{i}\left|\right|\leq \nu_{i},\;i=1,2,\ldots ,n,$$

where $\sigma_{i}$ denotes the *i*th component of σ and $\nu_{i}>0$ defines its prescribed admissible bound.

The control problem considered in this work can therefore be stated as follows: design linear and homogeneous feedback controllers for the magnetic-force input such that the magnetic microparticle tracks the prescribed reference trajectory while satisfying the state constraints in (23) and reducing the influence of the bounded external disturbance defined in (11) on the tracking output.

## 3. Preliminaries on Homogeneous Control and Invariant Sets

This section recalls the main concepts of homogeneous systems and invariant sets required for the development of the constrained control framework proposed in the subsequent sections. In particular, linear dilations, canonical homogeneous norms, homogeneous control design for linear systems, and homogeneous invariant ellipsoids are introduced.

### 3.1. Dilation and Canonical Homogeneous Norm

Homogeneity characterizes the symmetry of an object, such as a function, a vector field, or a set, with respect to a group of transformations referred to as dilations. In this work, only linear dilations are considered [20]. A linear dilation is defined by

(24) $$\sigma \mapsto d\left(s\right)\sigma ,\;\sigma \in R^{n},\;s\in R,$$

where *s* denotes the dilation parameter and

(25) $$d\left(s\right)=e^{sH_{d}}.$$

The anti-Hurwitz matrix $H_{d}\in R^{n\times n}$ is referred to as the generator of the dilation d.

The monotonicity property implies that d(s) acts as a contraction for s<0 and as an expansion for s>0. Consequently, for every $\sigma \in R^{n}\setminus \left\{0\right\}$, there exists a unique pair $\left(s_{0},\sigma_{0}\right)$ such that $\sigma =d\left(s_{0}\right)\sigma_{0}$, where $\sigma_{0}$ belongs to the corresponding unit sphere.

**Theorem** **1** ([21])**.** *The dilation d is strictly monotone in $R^{n}$ with respect to ${∥\cdot ∥}_{P}$ if and only if the following linear matrix inequality holds:*

(26) $$PH_{d}+H_{d}^{⊤}P≻0,\;P≻0,$$

*where $H_{d}\in R^{n\times n}$ is the generator of the dilation d.*

For a strictly monotone dilation, the canonical homogeneous norm can be introduced through a homogeneous projection onto the unit sphere [20,22].

**Definition** **1** ([20])**.** *Given a strictly monotone linear dilation d with respect to the norm ${∥\cdot ∥}_{P}$, the d-homogeneous function ${∥\cdot ∥}_{d,P}:R^{n}\mapsto \left[0,+\infty \right)$ defined by*

(27) $${∥\sigma ∥}_{d,P}:=\left\{\begin{array}{cc} e^{s_{\sigma }}:\; & ∥d\left(-s_{\sigma }\right){\sigma ∥}_{P}=1,if\;\sigma \neq 0,\\ 0,if\; & \sigma =0, \end{array}\right.$$

*is called the canonical homogeneous norm.*

The derivative of this norm is recalled in Appendix A.

Moreover, for any $\sigma \in R^{n}$ and s∈R, the canonical homogeneous norm satisfies

(28) $${∥\sigma ∥}_{d,P}={∥-\sigma ∥}_{d,P}{,\;∥d\left(s\right)\sigma ∥}_{d,P}=e^{s}{∥\sigma ∥}_{d,P},$$

and

(29) $${∥\sigma ∥}_{d,P}=1\;⟺\;{∥\sigma ∥}_{P}=1.$$

The standard definitions of homogeneous scalar functions and vector fields are recalled in Appendix B.

### 3.2. Homogeneous Control Design for Linear Systems

Homogeneous control systems can provide important performance properties compared with conventional linear feedback, including finite-time convergence for appropriate negative homogeneity degrees and enhanced robustness properties [20,23,24]. The following theorem recalls a generalized homogeneous control design procedure for linear systems and summarizes the results reported in [25,26,27].

**Theorem** **2.** *For the time-invariant controllable system*

(30) $$\dot{\sigma }=A\sigma +Bu,$$

*let the pair (A,B) be controllable. Then:*

*(1)*  *Any solution $Y_{0}\in R^{m\times n}$, $H_{0}\in R^{n\times n}$ of the linear algebraic equations*
(31) $$AH_{0}-H_{0}A+BY_{0}=A,\;H_{0}B=0$$
  *is such that $H_{0}-I_{n}$ is invertible, $H_{d}=I_{n}+\mu H_{0}$ is anti-Hurwitz, and*
(32) $$A_{0}=A+BY_{0}{\left(H_{0}-I_{n}\right)}^{-1}$$
  *satisfies*
(33) $$A_{0}H_{d}=\left(H_{d}+\mu I_{n}\right)A_{0},\;H_{d}B=B.$$
*(2)*  *The linear algebraic system*
(34) $$\begin{array}{cc} \; & A_{0}X+XA_{0}^{⊤}+BY+Y^{⊤}B+\rho \left(H_{d}X+XH_{d}^{⊤}\right)=0, \end{array}$$
(35) $$\begin{array}{cc} \; & H_{d}X+XH_{d}^{⊤}≻0,\;X=X^{⊤}≻0 \end{array}$$
  *has a solution $X\in R^{n\times n}$, $Y\in R^{m\times n}$ for any $\rho \in R_{+}$.*
*(3)*  *The canonical homogeneous norm ${∥\cdot ∥}_{d,P}$ induced by the weighted Euclidean norm ${∥\sigma ∥}_{P}=\sqrt{\sigma^{⊤}P\sigma }$, with $P=X^{-1}$, is a Lyapunov function for system *(30)* under the feedback law*
(36) $$\begin{array}{cc} u_{h}\left(\sigma \right)\; & =K_{0}\sigma +{∥\sigma ∥}_{d,P}^{1+\mu }{Kd(-\ln∥\sigma ∥}_{d,P})\sigma , \end{array}$$
(37) $$\begin{array}{cc} K_{0}\; & =Y_{0}{\left(H_{0}-I_{n}\right)}^{-1},\;K=YX^{-1}. \end{array}$$
*(4)*  *The closed-loop system *(30)* with controller *(36)* is uniformly finite-time stable.*

### 3.3. Homogeneous Invariant Ellipsoids

To account for bounded disturbances and state constraints, we recall the invariant ellipsoid for the tracking-error system (22) [14,28].

**Definition** **2.** *The ellipsoid*

(38) $$\varepsilon \left(P\right)=\left\{\sigma \in R^{n}:{∥\sigma ∥}_{P}\leq 1\right\},\;P≻0,$$

*is called a state invariant set for the tracking-error variable σ(t) of system *(22)* under the bounded disturbance ${∥\omega ∥}_{Q}\leq 1$ if*

(39) σ(0)∈ε(P)⟹σ(t)∈ε(P),∀t≥0.

Similarly, a d-homogeneous ellipsoid is defined as

(40) $$\varepsilon_{d}\left(P\right)=\left\{\sigma \in R^{n}:{∥\sigma ∥}_{d,P}\leq 1\right\},\;P≻0,$$

where ${∥\cdot ∥}_{d,P}$ denotes the canonical homogeneous norm induced by the weighted Euclidean norm ${∥\cdot ∥}_{P}$.

**Definition** **3** ([29])**.** *For system *(22)* under the bounded disturbance ${∥\omega ∥}_{Q}\leq 1$, a d-homogeneous ellipsoid $\varepsilon_{d}\left(P\right)$ with configuration matrix P is said to be:*

- *State* ***invariant*** *with respect to σ(t) if $\sigma \left(0\right)\in \varepsilon_{d}\left(P\right)$ implies $\sigma \left(t\right)\in \varepsilon_{d}\left(P\right)$ for all t≥0;*
- *State* ***attractive*** *with respect to σ(t) if $\sigma \left(0\right)\notin \varepsilon_{d}\left(P\right)$ implies that $\sigma \left(t\right)\to \varepsilon_{d}\left(P\right)$ as t→∞.*

By selecting an appropriate dilation d, a conventional ellipsoid can be represented as a d-homogeneous ellipsoid. Moreover, conventional and d-homogeneous invariant ellipsoids admit the same boundary characterization, which will be used in the subsequent controller design.

**Lemma** **1** ([29])**.** *The following two claims are equivalent:*

*1.*  *$\varepsilon_{d}\left(P\right)$ is a d-homogeneous invariant ellipsoid of the system *(22)*;*
*2.*  *$\sigma^{⊤}P\left(A\sigma +BF_{m}+G+D\omega \right)\leq 0$ for all $\sigma \in R^{n}:{∥\sigma ∥}_{P}=1$ and $\forall \omega \in R^{p}:{∥\omega ∥}_{Q}\leq 1$.*

The above results provide the mathematical tools required to construct linear and homogeneous feedback controllers while explicitly enforcing invariance of the prescribed tracking-error region in the presence of bounded disturbances.

## 4. Constrained Control and Optimal Disturbance Rejection

In this section, the invariant ellipsoid method is applied to design the linear and homogeneous controllers for system (22) under the state constraints (23) in order to track the reference trajectory while optimally rejecting the external disturbance. Using this approach, the optimization problem is reduced to finding the smallest invariant ellipsoid characterized by a set of linear matrix inequalities (LMIs). The resulting optimization problem can be solved numerically using suitable MATLAB R2020a optimization tools, such as YALMIP.

Definitions 2 and 3 show that any trajectory starting inside an invariant ellipsoid remains inside it for all subsequent time. This ellipsoid is therefore referred to as a state invariant set. The objective of the optimal controller design is to minimize the effect of the external disturbance on the system output.

It is therefore reasonable to introduce an invariant set associated with the output variable *y*. Similar to the state invariant set, the output invariant set for system (12)–(13) is defined as

(41) $$\varepsilon_{y}\left(P\right)=\left\{y\in R^{m}:y^{⊤}{\left(CP^{-1}C^{⊤}\right)}^{-1}y\leq 1\right\},\;P≻0.$$

In the ideal case without external disturbance, the output of system (22) is able to converge to the origin. However, when the external disturbance ω is considered, the system output converges to an invariant set under an optimal controller. Therefore, the output invariant ellipsoid $\varepsilon_{y}\left(P\right)$ can be used to characterize the effect of the external disturbance.

Minimizing the influence of the exogenous disturbance ω on the output *y* consequently requires minimizing the output invariant ellipsoid $\varepsilon_{y}\left(P\right)$, which is characterized by the matrix $CP^{-1}C^{⊤}$. In this work, the trace of this matrix is used as a measure of the size of the output invariant ellipsoid. Therefore, the disturbance rejection objective is formulated as

(42) $$\mathrm{tr}(CP^{-1}C^{⊤})\;⟶\;\mathrm{Min}.$$

### 4.1. Linear Controller with State Constraint

In the following theorem, we first introduce the optimized linear controller for system (22). The controller stabilizes the system and rejects the external disturbance while minimizing the output invariant ellipsoid.

**Theorem** **3.** *Let $P=P^{⊤},X=P^{-1}\in R^{6\times 6},Y\in R^{3\times 6}$ be the optimal solution of*

(43) $$\begin{array}{c} tr(CP^{-1}C^{⊤})\;\to \;Min \end{array}$$

*under constraint*

(44) $$W\;:=\left(\begin{array}{cc} AX\;+\;XA^{⊤}\;+\;BY\;+\;Y^{⊤}\;B^{⊤}\;+\;\eta X & D\\ D^{⊤} & -\eta Q \end{array}\right)⪯\;0$$

(45) $$\left(\begin{array}{cc} X & X^{⊤}e_{i}^{⊤}\\ e_{i}X & \nu^{2} \end{array}\right)⪰0$$

*then the optimized linear controller*

(46) $$u=K_{\mathrm{lin}}\sigma .$$

(47) $$K_{lin}=YX^{-1},\;B_{0}=\left[0,mI\right],\;H_{0}=-G$$

*is able to drive system *(22)* under constraint *(23)* to a state invariant set ε(P) while minimizing the output invariant ellipsoid $\varepsilon_{y}\left(P\right)$.*

**Proof.**  Let $V\left(\sigma \right)=\sigma^{⊤}P\sigma$ and $X=P^{-1}$. For $u=K_{\mathrm{lin}}\sigma$, the S-procedure applied to the disturbance bound and the Schur complement yield the invariance LMI (44). The Schur complement of LMI (45) gives $e_{i}^{⊤}e_{i}⪯\nu_{i}^{2}P$. Consequently, $\sigma_{i}^{2}\leq \nu_{i}^{2}V\left(\sigma \right)\leq \nu_{i}^{2}$ for σ∈ε(P). Detailed algebra is provided in Appendix C. □

The main idea in the proof of Theorem 3 is to guarantee that the Lyapunov function satisfies $\dot{V}\left(x\right)\leq 0$ for V(x)≥1 in the presence of bounded disturbances and state constraints. It is therefore important to determine the admissible disturbance that maximizes $\dot{V}\left(x\right)$. This disturbance is referred to as the worst-case disturbance.

**Corollary** **1.** *The worst-case disturbance $\bar{\omega }$ for system *(22)* with controller *(46)* is given by*

(48) $$\bar{\omega }=\frac{D^{⊤}P\sigma }{∥D^{⊤}P\sigma ∥}$$

**Proof.**  The term depending on ω in $\dot{V}$ is $2\omega^{⊤}D^{⊤}P\sigma$. Its maximum over the unit Euclidean ball is attained in the direction $D^{⊤}P\sigma$, which gives the stated expression when $D^{⊤}P\sigma \neq 0$. At zero, every admissible disturbance maximizes this term. □

### 4.2. Homogeneous Controller with State Constraint

In this subsection, a homogeneous controller is introduced for system (22) to ensure convergence toward a d-homogeneous invariant/attractive ellipsoid.

**Theorem** **4.** *Let the controller u be defined as*

(49) $$u=u_{h}\left(\sigma \right)$$

*where $u_{h}\left(\sigma \right)$ is defined in Theorem 2, for some $X\in R^{n\times n}$ and some dilation d in $R^{n}$. Let $d_{\omega }=e^{sH_{d,\omega }}$ be a dilation in $R^{p}$ with $H_{d,\omega }=I_{p}+\mu \left(I_{p}+H_{0,\omega }\right)$ such that*

(50) $$H_{0}D=DH_{0,\omega }$$

*and *(31)* hold. If there exist β>0 and μ∈R such that*

(51) $$\begin{array}{cc} \; & W\;:=\;\left[\;\begin{array}{cc} A_{0}X\;+\;XA_{0}^{⊤}\;+\;BY\;+\;Y^{⊤}\;B^{⊤}\;+\;\beta X & D\\ D^{⊤} & -\beta Q \end{array}\;\right]\;⪯\;0 \end{array}$$

(52) $$\begin{array}{cc} \; & \left(\begin{array}{cc} X & X^{⊤}e_{i}^{⊤}\\ e_{i}X & \nu_{i}^{2} \end{array}\right)⪰0 \end{array}$$

*hold with $Y=KX\in R^{m\times n}$, and there exists μ∈R such that*

(53) $$\begin{array}{cc} \; & 1+\mu \lambda_{\min}\left(X^{-\frac{1}{2}}H_{0}X^{\frac{1}{2}}+X^{\frac{1}{2}}H_{0}^{⊤}X^{-\frac{1}{2}}\right)>0, \end{array}$$

(54) 1+μ+μRe(λi(H0,ω))>0

*then the d-homogeneous ellipsoid $\varepsilon_{d}\left(X^{-1}\right)$ is* ***invariant*** *for system *(22)*, *(49)* under the state constraint *(23)* and the bounded disturbance *(11)*.*

**Proof.**  The compatibility condition (50) makes the extended state–disturbance dynamics homogeneous under the specified dilations. Conditions (53) and (54) ensure their required monotonicity. On the common unit boundary ${∥\sigma ∥}_{d,P}={∥\sigma ∥}_{P}=1$, LMI (51) yields the inward-pointing condition of Lemma 1. The Schur complement of LMI (52) then bounds each component of σ by $\nu_{i}$ on the invariant set. The intermediate steps are given in Appendix E. □

Inspired by [28] and Theorem 4, the optimal tuning of the homogeneous controller (36) can be formulated in terms of the following Semi-Definite Programming (SDP) problem:

(55) $$\mathrm{tr}(CP^{-1}C^{⊤})\;\to \;Min$$

subject to the LMI constraints (51)–(52).

In the linear case, the invariant set is also an attractive set. Therefore, a similar result is established for the homogeneous case in the following corollary.

**Corollary** **2.** *If the conditions of Theorem 4 and the following LMI hold,*

(56) $$QH_{d_{\omega }}+H_{d_{\omega }}^{⊤}Q≻0,\;H_{d_{\omega }}⪰0,$$

*then the d-homogeneous invariant ellipsoid $\varepsilon_{d}\left(X^{-1}\right)$ is attractive under the state constraint.*

**Proof.**  For ${∥\sigma ∥}_{d,P}>1$, condition (56) bounds the dilated disturbance within its admissible set. Combined with LMI (51), this makes the canonical homogeneous norm decrease outside the unit set. The derivative calculation is in Appendix F. □

Inspired by Corollary 1, the worst-case disturbance for the optimal homogeneous controller is given in the following corollary.

**Corollary** **3.** *The worst-case disturbance $\bar{\omega }$ for system *(12)*–*(13)* with controller *(46)* is given by*

(57) $${\bar{\omega }}^{⊤}=\frac{Q^{-\frac{1}{2}}\sigma^{⊤}d^{⊤}{(-\ln∥\sigma ∥}_{d,P})Pd(-{\ln∥\sigma ∥}_{d,P})DQ^{-\frac{1}{2}}}{∥\sigma^{⊤}d^{⊤}{(-\ln∥\sigma ∥}_{d,P})Pd(-{\ln∥\sigma ∥}_{d,P})DQ^{-\frac{1}{2}}∥}$$

**Proof.**  The worst-case direction maximizes the term linear in ω in the derivative of the canonical homogeneous norm under ${∥\omega ∥}_{Q}=1$. The resulting normalized direction is stated above; see Appendix G. □

## 5. Simulation Results

### 5.1. Simulation Setup

In this simulation, a magnetic microparticle is considered to move in a blood-flow environment. The blood velocity is assumed to be constant and is given by

(58) $$v_{f}={\left[0.01,0.008,0.012\right]}^{⊤}\;m/s.$$

The other simulation parameters are summarized in Table 1.

Using these parameters, the state-space matrices are obtained as

(59) $$\begin{array}{c} A=\left(\begin{array}{cccccc} 0 & 0 & 0 & 1 & 0 & 0\\ 0 & 0 & 0 & 0 & 1 & 0\\ 0 & 0 & 0 & 0 & 0 & 1\\ 0 & 0 & 0 & -30 & 0 & 0\\ 0 & 0 & 0 & 0 & -30 & 0\\ 0 & 0 & 0 & 0 & 0 & -30 \end{array}\right),\;B=10^{6}\cdot \left(\begin{array}{ccc} 0 & 0 & 0\\ 0 & 0 & 0\\ 0 & 0 & 0\\ 2.12 & 0 & 0\\ 0 & 2.12 & 0\\ 0 & 0 & 2.12 \end{array}\right). \end{array}$$

The objective of the simulation is to demonstrate that the magnetic microparticle is able to track the reference trajectory defined by (17)–(18) while minimizing the effect of the bounded external disturbance on the output and satisfying the prescribed state constraints. Moreover, the simulation is used to compare the tracking performance of the linear and homogeneous controllers when the same optimized feedback gain is employed.

Assume that the matrix *C* is defined as

(60) $$C=\left(\begin{array}{cccccc} 0 & 0 & 0 & 0 & 0 & 0\\ 0 & 1 & 0 & 0 & 0 & 0\\ 0 & 0 & 1 & 0 & 0 & 0 \end{array}\right).$$

Then, the objective function to be optimized is

(61) $$\mathrm{tr}(CP^{-1}C^{⊤})\;\to \;Min.$$

Suppose that system (22) is considered with the following parameters:

(62) $$\begin{array}{cc} \; & \nu_{i}=0.07,\;\omega ={\left[1,1,1\right]}^{⊤}\cdot \cos\left(t\right), \end{array}$$

(63) $$\begin{array}{cc} \; & e_{i}=\left[1,1,1,0,0,0\right],\;Q=\left(\begin{array}{ccc} 1 & 0 & 0\\ 0 & 1 & 0\\ 0 & 0 & 1 \end{array}\right). \end{array}$$

### 5.2. Comparison of Linear and Homogeneous Controllers

The optimized linear controller is obtained by solving the SDP problem defined by (43)–(45). The optimized minimal invariant set of system (22) is characterized by the following matrix $P^{*}$:

(64) $$P^{*}=\left(\begin{array}{cccccc} 49.8093 & 42.7449 & 42.7449 & 6.0711 & 3.0336 & 3.0336\\ 42.7449 & 49.8093 & 42.7449 & 3.0336 & 6.0711 & 3.0336\\ 42.7449 & 42.7449 & 49.8093 & 3.0336 & 3.0336 & 6.0711\\ 6.0711 & 3.0336 & 3.0336 & 4.1885 & 0.0704 & 0.0704\\ 3.0336 & 6.0711 & 3.0336 & 0.0704 & 4.1885 & 0.0704\\ 3.0336 & 3.0336 & 6.0711 & 0.0704 & 0.0704 & 4.1885 \end{array}\right).$$

The optimized feedback gain of the linear controller is

$$K^{*}=10^{-4}\cdot \left(\begin{array}{cccccc} -0.0820 & -0.0609 & -0.0608 & 0.1183 & -0.0008 & -0.0008\\ -0.0714 & -0.0872 & -0.0661 & -0.0080 & 0.1182 & -0.0009\\ -0.0766 & -0.0766 & -0.0925 & -0.0082 & -0.0082 & 0.1180 \end{array}\right)$$

From Theorems 3 and 4, it can be observed that the design of the optimized homogeneous controller introduces only two additional conditions, namely (53) and (54), compared with the linear case.

As the homogeneous degree satisfies μ→0, conditions (53) and (54) can be satisfied. This indicates the existence of a feasible value of μ satisfying these conditions. To provide a consistent comparison between the linear and homogeneous controllers, the optimized parameters $P^{*}$ and $K^{*}$ obtained for the linear controller are also used for the homogeneous controller. A feasible value of μ is then selected such that (53), (54), and (56) are satisfied. Further details on upgrading a linear controller to its homogeneous counterpart can be found in [30].

The feasible homogeneous degree μ and dilation generator $H_{d}$ used in the simulation are

(65) $$\mu =-0.5,\;H_{d}=\left(\begin{array}{cccccc} 1.5 & 0 & 0 & 0 & 0 & 0\\ 0 & 1.5 & 0 & 0 & 0 & 0\\ 0 & 0 & 1.5 & 0 & 0 & 0\\ 0 & 0 & 0 & 1 & 0 & 0\\ 0 & 0 & 0 & 0 & 1 & 0\\ 0 & 0 & 0 & 0 & 0 & 1 \end{array}\right).$$

The initial state of the reference trajectory and the reference input are chosen as

(66) $$\xi_{r}\left(0\right)={\left(\begin{array}{cccccc} 0 & 0.2 & -0.4 & 0 & 0 & -0.7 \end{array}\right)}^{⊤},$$

and

(67) $$u_{0}=-{\left(\begin{array}{ccc} 10^{-6}, & 0, & 10^{-4} \end{array}\right)}^{⊤}.$$

The initial state of system (12) is

(68) $$\xi \left(0\right)={\left(\begin{array}{cccccc} 0.07 & 0.1 & 0.15 & 0 & 0 & 0 \end{array}\right)}^{⊤}.$$

The initial state of the error system (22) lies outside the invariant ellipsoid since

(69) $${\left(\xi \left(0\right)-\xi_{r}\left(0\right)\right)}^{⊤}P^{*}\left(\xi \left(0\right)-\xi_{r}\left(0\right)\right)>1.$$

**Figure 2:**
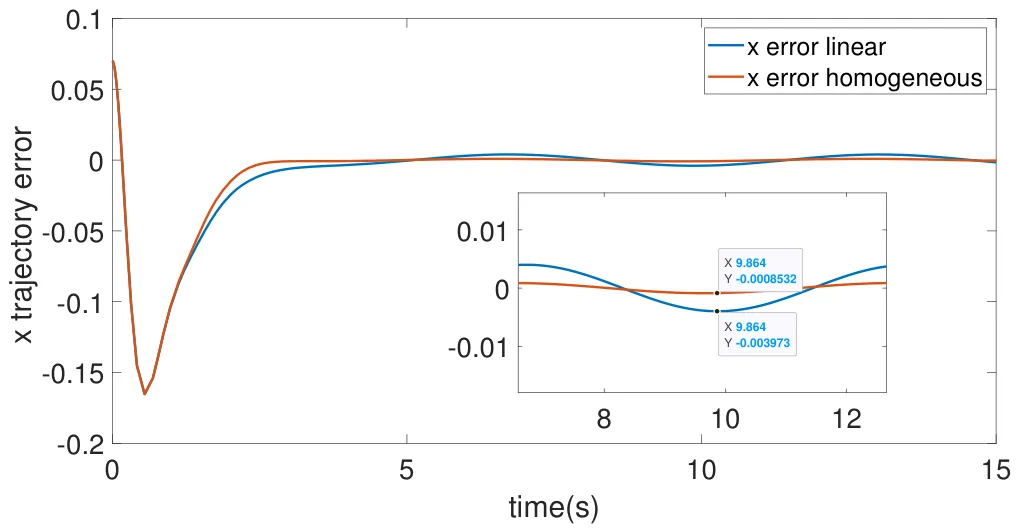
Comparison of the tracking error along the *x* direction.

**Figure 3:**
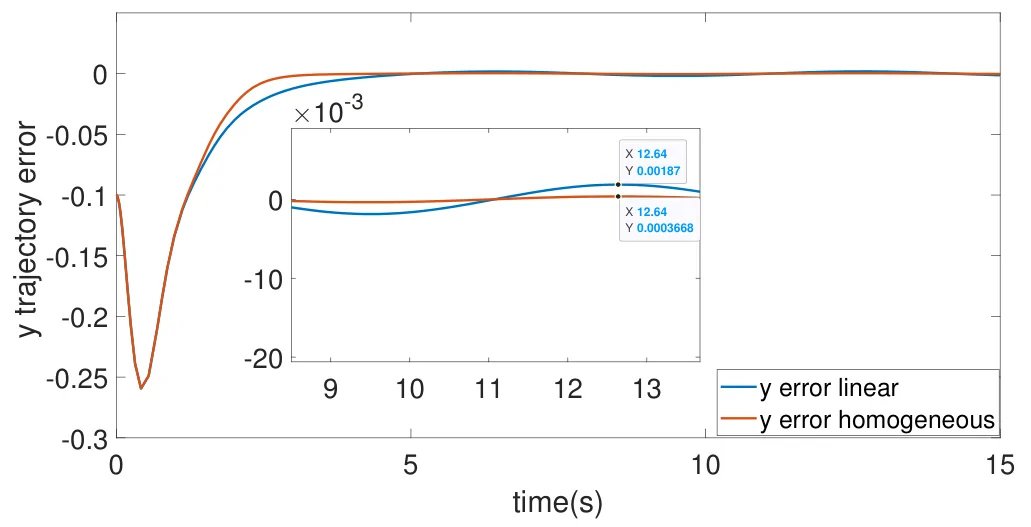
Comparison of the tracking error along the *y* direction.

**Figure 4:**
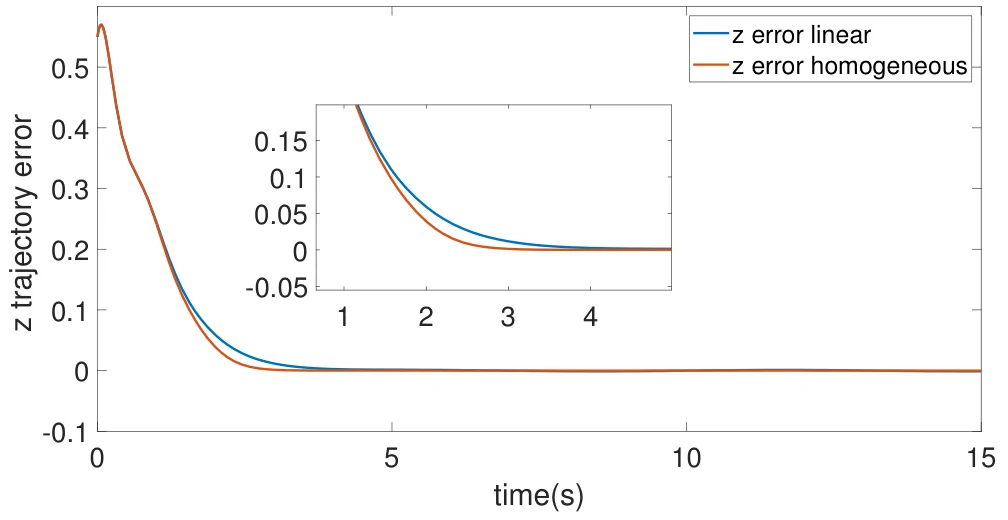
Comparison of the tracking error along the *z* direction.

The comparison of the ${\left[x,y,z\right]}^{⊤}$ tracking errors obtained using the linear and homogeneous controllers is presented in Figure 2, Figure 3 and Figure 4. The results show that the homogeneous controller provides faster convergence and improves the tracking accuracy along the *x*, *y*, and *z* directions, while using the same optimized feedback gain $K^{*}$ as the linear controller.

### 5.3. Discussion of Simulation Results

Figure 2, Figure 3 and Figure 4 show that both control strategies reduce the tracking errors in the three spatial directions despite the bounded time-varying disturbance. Since the linear and homogeneous controllers use the same optimized matrices $P^{*}$ and $K^{*}$, the comparison highlights the effect of the homogeneous formulation. The homogeneous controller provides faster convergence toward the prescribed invariant region and improves the trajectory-tracking behavior compared with the linear controller.

Table 2 extends the validation beyond the single condition shown in Figure 2, Figure 3 and Figure 4. For all twelve combinations of initial position error and disturbance amplitude, homogeneous feedback achieved a lower total integrated absolute tracking error than linear feedback using the published gains. The reduction ranged from 44.56% to 79.84%. Across these simulations, the largest sampled position error was 0.03 m, the largest sampled value of $\sigma^{T}P^{*}\sigma$ was 0.36531, and the largest commanded force norm was 7.019 μN. Thus, the sampled trajectories remained below the stated 0.07 m position-error threshold and within the unit ellipsoid, while the modeled force command remained below the 1 mN limit under these tested conditions. These observations do not constitute a general constraint certificate for all states inside that ellipsoid or verify force realizability by a specific permanent-magnet geometry.

We also repeated the same twelve scenarios using a second feasible LMI gain set designed with separate position-error constraints. Homogeneous feedback reduced the total IAE relative to linear feedback in every scenario for this second gain set as well, with reductions from 33.4% to 56.8%. This comparison provides evidence that the observed tracking improvement persists for the two tested designs; it does not establish robustness to arbitrary controller gains. Since the two designs use different *P* matrices, their *V* values should be interpreted relative to their respective ellipsoids rather than compared directly.

The simulation shown in Figure 2, Figure 3 and Figure 4 is initialized outside the invariant ellipsoid, showing the attractive behavior established theoretically. These results support the use of the proposed homogeneous extension for constrained magnetic microparticle navigation. Since the control design is formulated at the magnetic-force level, it can be considered for different permanent-magnet configurations provided that the commanded magnetic force can be generated within the actuator capabilities.

## 6. Conclusions

This paper investigated constrained trajectory tracking of magnetic microparticles actuated by permanent-magnet systems in the presence of bounded disturbances. A force-level formulation was adopted to describe the magnetic actuation independently of the specific permanent-magnet configuration.

An invariant-ellipsoid-based linear controller was first designed using LMIs to reduce the effect of bounded disturbances while satisfying prescribed state constraints. The design was then extended using homogeneous control. Simulation results showed that, using the same optimized feedback parameters, the homogeneous controller provides faster tracking-error convergence than the linear controller while preserving the invariant-set properties.

Future work will address the physical realization of the commanded magnetic forces. The analytical expressions in Section 2.1 provide a forward model: they relate a given permanent-magnet configuration to the magnetic field and, through Equation (5), to the force acting on the microparticle. The present study does not solve the inverse problem of determining feasible magnet positions and orientations for each commanded force, nor does it impose a force bound derived from a specific actuator geometry. We therefore plan to develop an inverse actuation method that accounts for the available workspace and achievable forces. We will also investigate a hybrid platform combining permanent magnets with electromagnets, whose complementary field gradients could provide trajectory corrections when the permanent-magnet configuration alone cannot sufficiently approximate the commanded force. These extensions will require actuator-level and experimental validation.

## Figures and Tables

**Table 1:** Simulation parameters.

Parameters	Values
Blood viscosity (α)	$3\times 10^{-3}$ Ns/m^2^
Radius of particle (*r*)	$0.25\times 10^{-3}$ m
Density of particle (ρ)	7200 kg/m^3^
Mass of particle (m)	$4.71\cdot 10^{-7}$ kg
Density of blood ($\rho_{f}$)	994 kg/m^3^
Gravitational acceleration (*g*)	9.8 m/s^2^

**Table 2:** Tracking performance with the controller gains reported in the manuscript under different initial position errors and disturbance amplitudes.

*h* (m)	*a* (m/s^2^)	${\mathrm{IAE}}_{\mathrm{lin}}$ (m s)	${\mathrm{IAE}}_{\mathrm{hom}}$ (m s)	Reduction (%)	maxV	$\max|\sigma_{x,y,z}|$ (m)	$\max∥F_{m}∥$ (μN)
0.01	0	0.01117	0.00532	52.38	0.04773	0.01000	5.527
0.01	0.1	0.06835	0.01634	76.10	0.04255	0.01000	5.510
0.01	0.3	0.19751	0.04057	79.46	0.04059	0.01197	5.472
0.01	0.5	0.32661	0.06585	79.84	0.04550	0.01991	5.423
0.02	0	0.02225	0.01149	48.37	0.16236	0.02000	6.433
0.02	0.1	0.07554	0.02232	70.46	0.16236	0.02000	6.396
0.02	0.3	0.20114	0.04585	77.20	0.16236	0.02000	6.315
0.02	0.5	0.32983	0.07080	78.53	0.16236	0.02000	6.226
0.03	0	0.03353	0.01859	44.56	0.36531	0.03000	7.019
0.03	0.1	0.08584	0.02928	65.89	0.36531	0.03000	6.990
0.03	0.3	0.20525	0.05245	74.45	0.36531	0.03000	6.925
0.03	0.5	0.33396	0.07715	76.90	0.36531	0.03000	6.848

*h* denotes the initial tracking error on each position axis, with zero initial velocity error. The disturbance is $a{\left[1,1,1\right]}^{T}\cos\left(t\right)$. The IAE is the sum of the three position-error absolute integrals over 15 s. The maxima of $V=\sigma^{T}P^{*}\sigma$, absolute position error, and commanded force norm are taken over both controllers and the simulated time samples. Reduction $=100(1-{\mathrm{IAE}}_{\mathrm{hom}}/{\mathrm{IAE}}_{\mathrm{lin}})$.

## Data Availability

The data presented in this study are available from the corresponding author upon reasonable request.

## Appendix A. Additional Properties of the Canonical Homogeneous Norm

For a strictly monotone dilation, it has been established in [21] that ${∥\cdot ∥}_{d,P}$ is single-valued and continuous on $R^{n}$, and continuously differentiable on $R^{n}\setminus \left\{0\right\}$. Its derivative with respect to σ is given by

(A1) $$\frac{{\partial ∥\sigma ∥}_{d,P}}{\partial \sigma }={∥\sigma ∥}_{d,P}\frac{\sigma^{⊤}d^{⊤}{(-\ln∥\sigma ∥}_{d,P})Pd(-{\ln∥\sigma ∥}_{d,P})}{\sigma^{⊤}d^{⊤}{(-\ln∥\sigma ∥}_{d,P})PH_{d}{d(-\ln∥\sigma ∥}_{d,P})\sigma },\;\sigma \neq 0.$$

## Appendix B. Homogeneous Functions and Vector Fields

**Definition** **A1.**
*A function $h:R^{n}\to R$ is said to be d-homogeneous of degree ν if*

(A2)
$$h\left(d\left(s\right)\sigma \right)=e^{\nu s}h\left(\sigma \right),\;\forall \sigma \in R^{n}\setminus \left\{0\right\},\;\forall s\in R.$$

**Definition** **A2.**
*A vector field $f:R^{n}\to R^{n}$ is said to be d-homogeneous of degree ν∈R if*

(A3)
$$f\left(d\left(s\right)\sigma \right)=e^{\nu s}d\left(s\right)f\left(\sigma \right),\;\sigma \in R^{n}\setminus \left\{0\right\},\;\forall s\in R.$$

## Appendix C. Details of the Linear Invariance and State-Constraint Proof

**Proof.** Since the output invariant ellipsoid $\varepsilon_{y}\left(P\right)$ characterizes the effect of the external disturbance, minimizing this invariant set reduces the influence of the disturbance. Therefore, the following objective is considered:

(A4) $$tr(CP^{-1}C^{⊤})\;\to \;Min$$

Consider the Lyapunov function candidate $V\left(\sigma \right)=\sigma^{⊤}P\sigma$. The ellipsoid ε(P) is a state invariant set for system (22) if and only if

(A5) $$\dot{V}\left(\sigma \right)\leq 0,\;\forall V\left(\sigma \right)\geq 1,\forall {∥\omega ∥}_{Q}\leq 1$$

Thus, with controller (46),

$$\begin{array}{cc} \dot{V}\left(\sigma \right)= & \sigma^{⊤}\left(PA+PBK_{lin}\right)\sigma +\sigma^{⊤}\left(A^{⊤}P+K_{lin}^{⊤}B^{⊤}P\right)\sigma \\ \; & +\sigma^{⊤}PD\omega +\omega^{⊤}D^{⊤}P\sigma \end{array}$$

Denote $s=\left(\begin{array}{c} \sigma \\ \omega \end{array}\right)$. Then,

$$\begin{array}{c} \dot{V}\left(x\right)=s^{⊤}\left(\begin{array}{cc} PA+PBK_{lin}+A^{⊤}P+K_{lin}^{⊤}B^{⊤}P & PD\\ D^{⊤}P & 0 \end{array}\right)s \end{array}$$

The condition V(σ)>1 can be rewritten as

(A6) $$\begin{array}{c} \sigma^{⊤}P\sigma >1\;\Leftrightarrow \;s^{⊤}\left(\begin{array}{cc} -P & 0\\ 0 & 0 \end{array}\right)s\leq -1 \end{array}$$

Similarly, the bounded disturbance ${∥\omega ∥}_{Q}\leq 1$ is equivalent to

(A7) $$\begin{array}{c} \omega^{⊤}Q\omega \leq 1\;\Leftrightarrow \;s^{⊤}\left(\begin{array}{cc} 0 & 0\\ 0 & Q \end{array}\right)s\leq 1 \end{array}$$

Denote

(A8) $$\begin{array}{cc} \; & T_{1}=\left(\begin{array}{cc} PA+PBK_{lin}+A^{⊤}P+K_{lin}^{⊤}B^{⊤}P & PD\\ D^{⊤}P & 0 \end{array}\right) \end{array}$$

(A9) $$\begin{array}{cc} \; & T_{2}=\left(\begin{array}{cc} -P & 0\\ 0 & 0 \end{array}\right)\;T_{3}=\left(\begin{array}{cc} 0 & 0\\ 0 & Q \end{array}\right) \end{array}$$

Thus, the conditions in (A5) are equivalent to

(A10) $$\begin{array}{c} s^{⊤}T_{1}s\leq 0\;\forall s:s^{⊤}T_{2}s<-1,\;s^{⊤}T_{3}s\leq 1 \end{array}$$

Using the S-procedure, the condition in (A10) is equivalent to

(A11) $$\begin{array}{c} T_{1}-\eta T_{2}-\gamma T_{3}⪯0,\;\forall \eta ,\gamma ,\eta \geq \gamma >0 \end{array}$$

Thus, one obtains

(A12) $$\left(\begin{array}{cc} PA+PBK_{lin}+A^{⊤}P+K_{lin}^{⊤}B^{⊤}P+\eta P & PD\\ D^{⊤}P & -\gamma Q \end{array}\right)⪯0$$

Using the Schur complement, we obtain

(A13) $$PA+PBK_{lin}+A^{⊤}P+K_{lin}^{⊤}B^{⊤}P+\eta P+\frac{1}{\gamma }PDD^{⊤}P⪯0$$

Therefore, if the LMI (A13) is satisfied, *P* provides a matrix characterizing an invariant ellipsoid. Consider the following two sets $\Gamma_{1}$ and $\Gamma_{2}$:

$$\Gamma_{1}=\left\{P:PA+PBK_{lin}+A^{⊤}P+K_{lin}^{⊤}B^{⊤}P+\eta P+\frac{1}{\eta }PDD^{⊤}P⪯0,\eta >0\right\}$$

$$\Gamma_{2}=\left\{P:PA+PBK_{lin}+A^{⊤}P+K_{lin}^{⊤}B^{⊤}P+\eta P+\frac{1}{\gamma }PDD^{⊤}P⪯0,\eta \geq \gamma >0\right\}$$

Thus, we obtain $\Gamma_{2}\subseteq \Gamma_{1}$. Without loss of generality, we propose

$$\begin{array}{c} \gamma =\gamma_{max}=\eta \end{array}$$

which leads to the LMI

$$PA+PBK_{lin}+A^{⊤}P+K_{lin}^{⊤}B^{⊤}P+\eta P+\frac{1}{\eta }PDD^{⊤}P⪯0$$

Denote $X=P^{-1}$. By pre-multiplying and post-multiplying the inequality by $P^{-1}$, we obtain (44). Then, we need to show that the state constraint $∥\sigma_{i}∥\leq \nu_{i}$ is satisfied if V(σ)≤1. It follows that

$$\begin{array}{cc} \left(\begin{array}{cc} X & X^{⊤}e_{i}^{⊤}\\ e_{i}X & \nu_{i}^{2} \end{array}\right)⪰0 & \;\Leftrightarrow \;X^{⊤}e_{i}^{⊤}e_{i}X\leq \nu_{i}^{2}X\\ \; & \;\Leftrightarrow \;e_{i}^{⊤}e_{i}\leq \nu_{i}^{2}P\\ \; & \;\Leftrightarrow \;\sigma_{i}^{2}=\sigma^{⊤}e_{i}^{⊤}e_{i}\sigma \leq \nu_{i}^{2}\sigma^{⊤}P\sigma \leq \nu_{i}^{2} \end{array}$$

Thus, the state constraint $∥\sigma_{i}∥\leq \nu_{i}$ is satisfied. □

## Appendix D. Derivation of the Worst-Case Linear Disturbance

**Proof.** The worst-case disturbance pushes the system trajectory toward the boundary of the invariant set. Therefore, it is obtained by maximizing the derivative of the Lyapunov function:

(A14) $$\dot{V}\left(x\right)\;\to \;max$$

From Theorem 3, we have

$$\dot{V}\left(\sigma \right)=\sigma^{⊤}\left(PA+PBK+A^{⊤}P+K^{⊤}B^{⊤}P\right)\sigma +2\omega^{⊤}D^{⊤}P\sigma$$

Thus, it is necessary to maximize

(A15) $$\omega^{⊤}D^{⊤}P\sigma \to \;max$$

The problem becomes

(A16) $$\max_{(\omega ,\omega )=1}\left(\omega^{⊤},D^{⊤}P\sigma \right)$$

Therefore, the worst-case disturbance is

(A17) $$\bar{\omega }=\frac{D^{⊤}P\sigma }{∥D^{⊤}P\sigma ∥}$$

□

## Appendix E. Details of the Homogeneous Invariance Proof

**Proof.** We first prove that, under condition (50), the vector field

(A18) $$\tilde{f}\left(\sigma ,\omega \right)=\left(\begin{array}{c} A\sigma +Bu(\sigma )+D\omega \\ 0 \end{array}\right)$$

is $\tilde{d}$-homogeneous of degree μ with respect to the dilation

(A19) $$\tilde{d}\left(s\right)=\left(\begin{array}{cc} d(s) & 0\\ 0 & d_{\omega }\left(s\right) \end{array}\right),s\in R.$$

Clearly, the inequality 1+μ+μRe(λi(H0,ω))>0 implies that $H_{d,\omega }$ is anti-Hurwitz and $d_{\omega }=e^{sH_{d,\omega }}$ is a dilation in $R^{p}$. Moreover, $1+\mu \lambda_{\min}\left(X^{-\frac{1}{2}}H_{0}X^{\frac{1}{2}}+X^{\frac{1}{2}}H_{0}^{⊤}X^{-\frac{1}{2}}\right)>0$ implies that $H_{d}X\;+\;XH_{d}^{⊤}\;≻\;0$. From condition (50), we obtain $H_{0}^{i}D=DH_{0,\omega }^{i}$, where *i* is an integer and i≥0. Then, we have

(A20) $$e^{\mu H_{0}s}D=\sum_{i=0}^{+\infty }\frac{\mu^{i}s^{i}H_{0}^{i}}{i!}D=D\sum_{i=0}^{+\infty }\frac{\mu^{i}s^{i}H_{0,\omega }^{i}}{i!}=De^{\mu H_{0,\omega }s}$$

Thus, we obtain

(A21) $$e^{\mu s}d\left(s\right)D=Dd_{\omega }\left(s\right).$$

Since $H_{0}$ is defined in Theorem 2, we derive that

$$f\left(d\left(s\right)\sigma ,d_{\omega }\left(s\right)\omega \right)=Ad\left(s\right)\sigma +Bu\left(d\left(s\right)\sigma \right)+Dd_{\omega }\left(s\right)\omega =$$

$$e^{\mu s}d\left(s\right)\sigma +e^{\mu s}d\left(s\right)Bu\left(\sigma \right)+e^{\mu s}d\left(s\right)D\omega =e^{\mu s}d\left(s\right)f\left(\sigma ,\omega \right),$$

where f(σ,ω)=Aσ+Bu(σ)+Dω. Let us denote $P=X^{-1}$. From the first LMI of (51), we derive

(A22) $${\left(\begin{array}{c} \sigma \\ \omega \end{array}\right)}^{⊤}\;\left(\begin{array}{cc} PA_{0}\;+\;A_{0}^{⊤}P\;+\;PBK\;+\;K^{⊤}B^{⊤}P\;+\;\beta P & PD\\ D^{⊤}P & -\beta Q \end{array}\right)\left(\begin{array}{c} \sigma \\ \omega \end{array}\right)\;⪯\;0$$

which can be written as

$$\begin{array}{cc} \; & \sigma^{⊤}\left(PA_{0}+A_{0}^{⊤}P+PBK+K^{⊤}B^{⊤}P\right)\sigma +\sigma^{⊤}PD\omega \\ \; & +\omega^{⊤}D^{⊤}P\sigma \leq \beta \left(\omega^{⊤}Q\omega -x^{⊤}P\sigma \right). \end{array}$$

Since ${∥\sigma ∥}_{d,P}$ is continuous at t=0 and $\sigma \to \sigma_{0}$ as t→0, then for $\omega^{⊤}Q\omega \leq 1$ and $∥\sigma_{0}{∥}_{d,P}=1$, we derive

$$\begin{array}{cc} \; & {\left.\frac{{d∥\sigma \left(t\right)∥}_{d,P}}{dt}\right|}_{t=0^{+}}=\frac{\sigma_{0}^{⊤}\left(PA_{0}+A_{0}^{⊤}P+PBK+K^{⊤}B^{⊤}P\right)\sigma_{0}+\sigma_{0}^{⊤}PD\omega +\omega^{⊤}D^{⊤}P\sigma_{0}}{\sigma_{0}^{⊤}\left(PH_{d}+H_{d}^{⊤}P\right)\sigma_{0}}\leq 0\\ \; & \Rightarrow \frac{\sigma_{0}^{⊤}P\left(A_{0}\sigma_{0}+BK\sigma_{0}+D\omega \right)}{\sigma_{0}^{⊤}PH_{d}\sigma_{0}}\leq 0. \end{array}$$

Here, the equivalence $∥\sigma_{0}{∥}_{d,P}=1\Leftrightarrow {∥\sigma_{0}∥}_{P}=1$ and the LMI (53) are used. By Lemma 1, we conclude that $\varepsilon_{d}\left(P\right)$ is a d-homogeneous **invariant** ellipsoid of system (22). Then, we need to show that the state constraint $∥\sigma_{i}∥\leq \nu_{i}$ is satisfied if ${∥\sigma ∥}_{d,P}\leq 1$. Since ${∥\sigma ∥}_{d,P}\leq 1\Rightarrow {∥\sigma ∥}_{P}\leq 1$, we have

$$\begin{array}{cc} \left(\begin{array}{cc} X & X^{⊤}e_{i}^{⊤}\\ e_{i}X & \nu_{i}^{2} \end{array}\right)⪰0\; & \;\Leftrightarrow \;X^{⊤}e_{i}^{⊤}e_{i}X\leq \nu_{i}^{2}X\\ \; & \;\Leftrightarrow \;e_{i}^{⊤}e_{i}\leq \nu_{i}^{2}P\\ \; & \;\Leftrightarrow \;\sigma_{i}^{2}=\sigma^{⊤}e_{i}^{⊤}e_{i}\sigma \leq \nu_{i}^{2}\sigma^{⊤}P\sigma \leq \nu_{i}^{2}. \end{array}$$

where $e_{i}$ is the base such that $\sigma_{i}=e_{i}\sigma$. Thus, the state constraint $∥\sigma_{i}∥\leq \nu_{i}$ is satisfied. □

## Appendix F. Details of the Attractiveness Proof

**Proof.** Consider the canonical norm as the Lyapunov function of system (22). Then, we have

$$\begin{array}{c} \frac{d}{dt}{∥\sigma ∥}_{d,P}=\frac{{∥\sigma ∥}_{d,P}^{1+\mu }}{2\sigma_{s}^{⊤}PH_{d}\sigma_{s}}\left({\left(\begin{array}{c} \sigma_{s}\\ \omega_{s} \end{array}\right)}^{⊤}\Gamma \left(\begin{array}{c} \sigma_{s}\\ \omega_{s} \end{array}\right)-\beta +\beta \omega_{s}^{⊤}Q\omega_{s}\right) \end{array}$$

where $\sigma_{s}=d\left(-s\right)\sigma$, $\omega_{s}=d_{\omega }\left(-s\right)\omega$ with $s={\ln∥\sigma ∥}_{d,P}$ and

(A23) $$\Gamma =\left(\begin{array}{cc} PA_{0}+A_{0}^{⊤}P+PBK+K^{⊤}B^{⊤}P+\beta P & PD\\ D^{⊤}P & -\beta Q \end{array}\right)$$

From condition (51), one derives Γ⪯0. From condition (56), we obtain, for any s>0,

$$\begin{array}{cc} \int_{0}^{s}\frac{d}{d\tau }\omega_{s}^{⊤}Q\omega_{s}d\tau & =\int_{0}^{s}\frac{d}{d\tau }\omega_{s}^{⊤}\left(H_{d_{\omega }}+QH_{d_{\omega }}^{⊤}Q\right)\omega_{s}d\tau \\ \; & =\omega_{s}^{⊤}Q\omega_{s}-\omega^{⊤}Q\omega <0 \end{array}$$

Then, we have $\omega_{s}^{⊤}Q\omega_{s}<\omega^{⊤}Q\omega \leq 1$. Therefore, for any ${e:∥\sigma ∥}_{d,P}>1,\omega \neq 0$ and ∥ω∥≤1, we conclude that $\frac{d}{dt}{∥\sigma ∥}_{d,P}<0$. This implies that the d-homogeneous ellipsoid $\varepsilon_{d}\left(P\right)$ is attractive. □

## Appendix G. Derivation of the Worst-Case Homogeneous Disturbance

**Proof.** For the external disturbance to push the system trajectory toward the boundary of the d-homogeneous invariant ellipsoid, the following condition is considered:

(A24) $$\frac{{d∥\sigma ∥}_{d,P}}{dt}\;\to \;Max$$

where

$$\begin{array}{cc} \frac{{d∥\sigma ∥}_{d,P}}{dt}\; & =\frac{\sigma^{⊤}{d(-\ln(∥\sigma ∥}_{d,P}))Pd(-{\ln(∥\sigma ∥}_{d,P}))\left(A\sigma +Bu\left(\sigma \right)\right)}{\sigma^{⊤}d^{⊤}{(-\ln(∥\sigma ∥}_{d,P}))PH_{d}{d(-\ln(∥\sigma ∥}_{d,P}))\sigma }\\ \; & +\frac{\sigma^{⊤}{d(-\ln(∥\sigma ∥}_{d,P}))Pd(-{\ln(∥\sigma ∥}_{d,P}))D\omega }{\sigma^{⊤}d^{⊤}{(-\ln(∥\sigma ∥}_{d,P}))PH_{d}{d(-\ln(∥\sigma ∥}_{d,P}))\sigma }\leq 0 \end{array}$$

Thus, it is required to maximize

(A25) $$\sigma^{⊤}{d(-\ln(∥\sigma ∥}_{d,P}))Pd(-{\ln(∥\sigma ∥}_{d,P}))D\omega \to \mathrm{Max}$$

with ${∥\omega ∥}_{Q}=1$. Therefore, the worst-case disturbance is

$${\bar{\omega }}^{⊤}=\frac{Q^{-\frac{1}{2}}\sigma^{⊤}d^{⊤}{(-\ln∥\sigma ∥}_{d,P})Pd(-{\ln∥\sigma ∥}_{d,P})DQ^{-\frac{1}{2}}}{∥\sigma^{⊤}d^{⊤}{(-\ln∥\sigma ∥}_{d,P})Pd(-{\ln∥\sigma ∥}_{d,P})DQ^{-\frac{1}{2}}∥}$$

□
